# Medical facemask waste alters detritus decomposition and fungal communities in a freshwater pond

**DOI:** 10.1038/s41598-026-45795-5

**Published:** 2026-03-30

**Authors:** Ze Hui Kong, Martina Stangl, Rebecca Oester, Svante Rehnstam, Martyn Futter, Abu Bakar Siddique, Mirco Bundschuh, Brendan G. Mckie

**Affiliations:** 1 Department of Aquatic Sciences and Assessment, Swedish University of Agricultural Sciences, Uppsala, Sweden; 2 Tvärminne Zoological Station, Faculty of Biological and Environmental Sciences, University of Helsinki, Hanko, Finland; 3 Department of Environment and Biodiversity, University of Salzburg, Salzburg, Austria; 4 Institute of Microbiology, University of Applied Sciences and Arts of Southern Switzerland, Mendrisio, Switzerland; 5 Department of Evolutionary Biology and Environmental Studies, University of Zurich, Zurich, Switzerland; 6 Department of Aquatic Ecology, Swiss Federal Institute of Aquatic Science and Technology, Dübendorf, Switzerland; 7 Department of Plant Biology, Swedish University of Agricultural Sciences, Uppsala, Sweden; 8 Institute for Environmental Sciences, iES Landau, University of Kaiserslautern-Landau (RPTU), Landau, Germany

**Keywords:** Leaf litter decomposition, Cotton tensile strength loss, Aquatic fungi, Macroplastic, Microplastic, Plastic leachates, Ecology, Ecology, Environmental sciences

## Abstract

Plastic pollution is an ongoing issue in freshwater ecosystems, including that generated from the spike in disposable facemask use during the COVID-19 pandemic. The degradation products of such plastic waste, including plastic leachate compounds and generation of microplastics, have the potential to affect freshwater ecosystem structure and function. We investigated the effects of facemask-derived polypropylene particles of different sizes and their leachates on fungal communities and detritus decomposition in a pond. We further investigated effects of the presence of wood shavings, used to represent a naturally-occurring, highly refractory, organic reference material. Over five weeks, leaf litter mass loss and cotton cellulose tensile strength loss were quantified weekly, and fungal biomass, community composition, and functional gene abundance at two time points. Wood shavings reduced leaf decomposition (-4.4%) relative to controls, while plastics increased decomposition of labile cotton cellulose (+ 6.6%), with the strongest effect from unleached microplastics (+ 22.7%). After 21 days, litter-associated fungal biomass was reduced by the presence of wood shavings (-20.1%) and plastics (-8.6%). Fungal communities differed between wood- and control treatments, and varied widely under plastic exposure. Our findings highlight size- and leachate-dependent effects of facemask-derived plastic particles on freshwater fungal communities and ecosystem functions, which largely contrasted with those of wood.

**Supplementary Information:**

The online version contains supplementary material available at 10.1038/s41598-026-45795-5.

## Introduction

The continuing rise in plastic production^1,2^ is expected to increase the volume of plastic waste in the environment. A recent example is provided by the extensive production and use of disposable personal protective equipment – particularly disposable polypropylene (PP) medical facemasks^3,4^ (FMs) – during the COVID-19 pandemic. The improper disposal of FM waste made it ubiquitous in the environment^5^, including freshwaters^6,7^. The risk of future epidemics driven by respiratory illness and associated facemask mandates^8,9^, together with ongoing widespread public facemask use in some regions of the world^10^, highlight the need to understand not only the environmental impacts of PP particles per se, but also of the particular types of particles generated from facemask waste. Macroplastic (⌀>5000 μm) waste breaks down in the environment and releases microplastics (⌀=1–5000 μm) over time, with physical, chemical and biological processes driving the rate of microplastic generation^11,12^. FM-derived waste is particularly notable for easily releasing microplastic sheets, fibres and fragments^13^, sometimes within as little as 24 h of being submerged^14^. The influx and transport of both macro- and microplastics in freshwater ecosystems may threaten biodiversity and ecosystem functioning^15^. Impacts of microplastics have received significant research attention in recent years, with varying effects on ecosystem functioning detected^16–21^. However, most freshwater studies have been conducted in indoor laboratory-based systems (but see^19,22–24^ which used field-based mesocosms), and research on impacts of macroplastic pollution in freshwater ecosystems is limited^25–27^. Accordingly, there is an urgent need to advance our understanding of how macro- and microplastic waste affects key organisms and the essential ecological functions they regulate in freshwaters, particularly under more complex field conditions^28,29^.

Among the key freshwater ecosystem processes vulnerable to anthropogenic pollution is the decomposition of terrestrial plant detritus (e.g. leaf litter). Leaf litter decomposition is driven by decomposer microorganisms that contribute to water purification, nutrient cycling and the assimilation of terrestrial nutrients into aquatic food webs^30–32^. These microorganisms include bacteria and aquatic hyphomycete fungi, which decompose both labile and refractory litter components^30,33^. Fungi are primary agents of microbial-mediated decomposition in freshwater habitats^34,35^, producing a wide array of extracellular enzymes that target structural polysaccharides in plant cell walls^36,37^, with some taxa able to degrade highly refractory woody substrates^38^. In contrast, bacteria typically produce enzymes at lower levels compared to fungi^39–41^, and thus normally contribute a smaller fraction of total detritus decomposition^42,43^. The activities of fungi and other microorganisms on leaf litter enhance its palatability for detritivore “shredder” invertebrates (a process described as *conditioning*^44,45^, which in turn drive mechanical litter fragmentation^34,46^. Disruptions to fungal community composition, growth and activity can therefore have consequences for nutrient cycling and subsequent use of leaf litter as a resource by other organisms in freshwater ecosystems^44,47^.

In aquatic ecosystems, leaf litter, inorganic particles and woody detritus (e.g. twigs) accumulate into litter packs^48,49^, which increasingly also include anthropogenic plastic waste^50^. The presence of highly refractory woody materials can slow leaf decomposition, either by physically hindering the spread of fungal hyphae^51,52^ or by releasing natural inhibitory compounds, e.g. tannins^53^. The presence of highly refractory plastic debris in leaf packs might similarly interfere with microbial-mediated decomposition. Freshly discarded plastics release leachates that may contain toxic additives such as plasticizers, dyes and softeners^54,55^, potentially affecting fungi and their enzyme activity^56^. Furthermore, the distinct material and surface properties of plastics might cause effects that differ from those of natural refractory materials. For example, plastic surfaces may provide novel substrates for microbial colonization in freshwater environments^57,58^, potentially supporting fungal communities distinct from those found in the water column or on submerged wood^59^, with the potential to alter microbial-mediated ecosystem functions^60^.

**Fig. 1:**
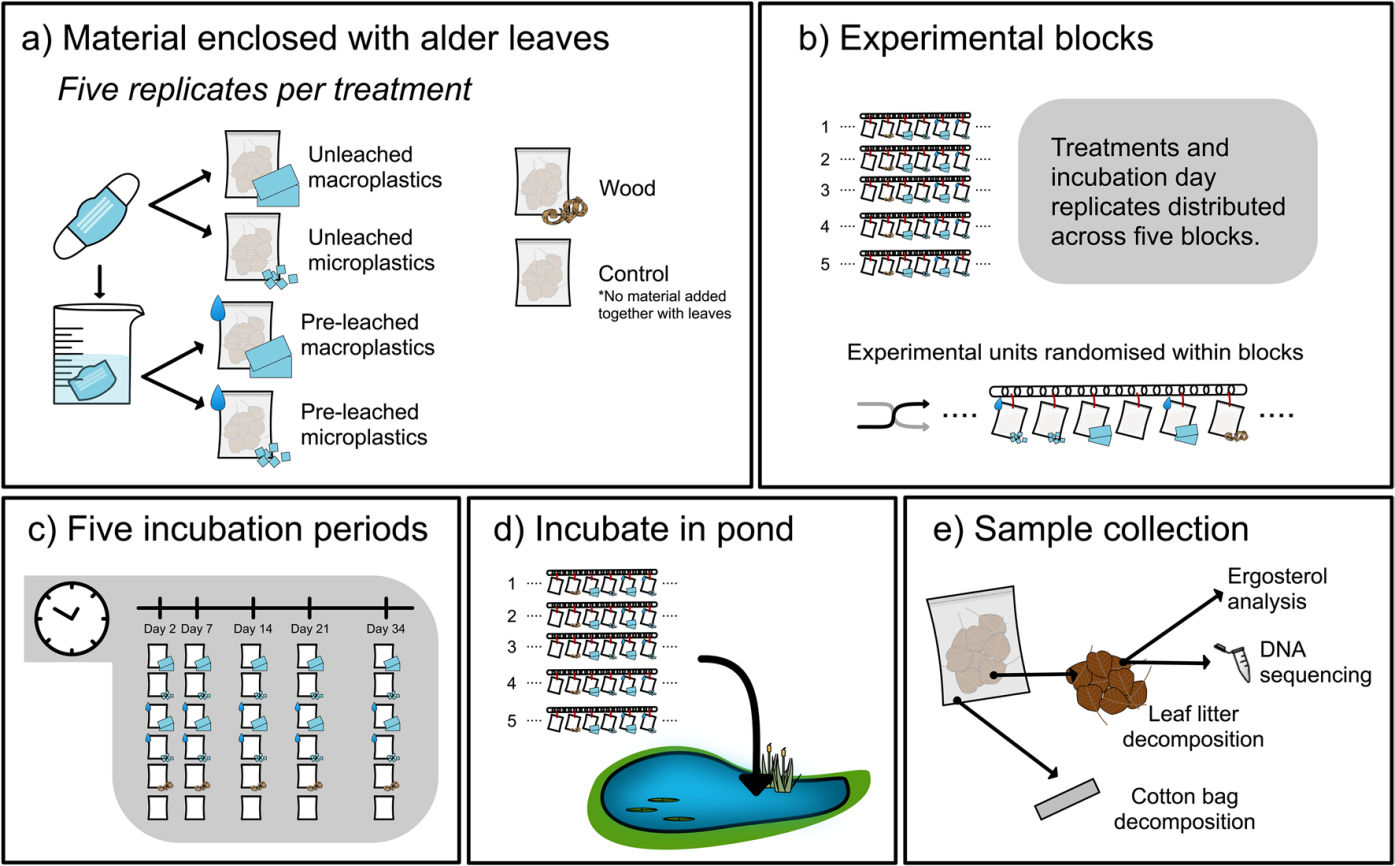
Summary of experimental methods used in this study. (**a**) Alder leaf litter was enclosed in cotton mesh bags, and allocated to one of six treatments, including controls (no additional material), or with addition of wood shavings or plastic particles. The plastic exposure treatments further varied in particle size and leaching status. (**b**) The six experimental treatments are crossed with five incubation periods in one experimental block. These five replicates were evenly distributed across the five experimental blocks, i.e. one replicate per block. All experimental units were randomly distributed within each experimental block, which comprised the stainless-steel chains litterbags are attached to. (**c**) Example of one experimental block: encompassing five replicates for each treatment and incubation period combination. (**d**) All bags were incubated in the pond. (**e**) Bags were collected after each incubation period and the illustrated variables quantified. Ergosterol analysis and DNA sequencing were only conducted for samples from two of the five incubation periods, at day 2 and 21.

We conducted a field investigation to assess the effects of macro- and microplastics derived from disposable PP-FMs on microbial-mediated detritus decomposition and fungal communities. Fine mesh cotton bags (mesh size: 0.5 mm) were deployed in a pond, containing either alder (*Alnus glutinosa*) leaf litter only, leaf litter with plastic particles, or leaf litter with mixed conifer wood shavings (Fig. 1). The wood shavings served as a surrogate for naturally occurring, highly refractory, organic matter. We additionally varied plastic particle size, macro- (60 × 60 mm) or micro- (3 × 3 mm) plastics, and leaching status (leached versus unleached). At five time points across five weeks (Fig. 1c), we quantified mass loss of the alder litter to assess decomposition of a naturally-occurring detrital substrate, and measured tensile strength loss (TSL) of cotton bags to assess decomposition of cellulose, a labile Carbon compound, following a standard cotton-strip assay-type approach^61,62^. In both cases, we expect decomposition to primarily reflect the activities of microbial organisms, given the bag mesh size is sufficiently small to exclude most invertebrates from accessing the leaf litter, and breakdown of cotton material in freshwaters is primarily driven by microbial catabolism^63^. Additionally, we assessed fungal biomass, community composition and functional gene abundance on leaf litter at the start and end of the experiment. The chemical composition of wood and plastic leachates was also characterized with high-resolution mass spectrometry. Given the potential for both plastic and refractory organic matter to interfere with microbial activity and detritus decomposition due to leachate toxicity or physical obstruction, we hypothesised that: (H1) the presence of refractory materials (plastics or wood shavings) reduces decomposition, (H2) larger-sized plastic particles are associated with a greater reduction in decomposition, due to their greater potential to impose a barrier to fungal growth, and (H3) unleached plastics more strongly inhibit decomposition than leached plastic.

## Results

Primary tests of significance were obtained from linear mixed-effects models. Further tests were done using log response ratios to evaluate variation between treatments and controls. Percentage changes in mean values are reported as effect sizes.

### Leaf mass loss

**Fig. 2:**
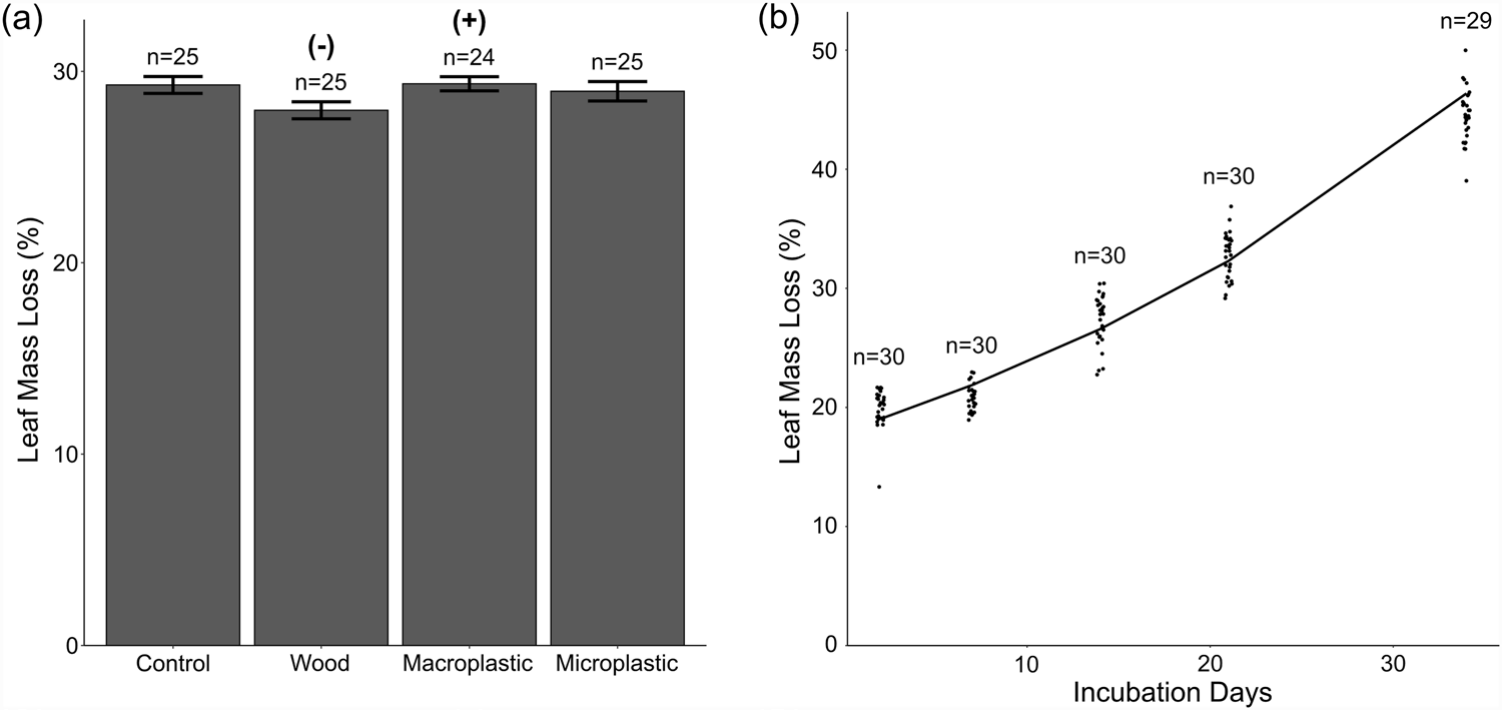
(**a**) Mean (± SE) leaf mass loss when exposed to wood or either macro- or microplastics, averaged across all sampling dates (2, 7, 14, 21 and 34 days). Statistically significant differences between material addition treatments and controls identified using log response ratios (Supplementary Fig. S1) are denoted with (+) and (-) to indicate positive or negative effect sizes respectively. (**b**) Leaf mass loss over time, pooling across all experimental treatments. Lines represent predicted trends based on the mixed model.

Leaf mass loss was affected by particle size nested in material (Fig. 2a; F_1,133_ = 5.89, *p* = 0.017), but the effect size was small, with macroplastics associated with 0.3% higher mass loss relative to controls. Additionally, log response ratios indicate that exposure to wood shavings was associated with 4.4% lower mass loss relative to controls (Fig. 2a, Supplementary Fig. S1). Generally, leaf mass loss increased through time (F_1,133_= 2301, *p* < 0.001), with a 124% increase between 2 and 34 days of incubation (Fig. 2b). There were no significant interactions between time and the treatments (*p* > 0.050).

### Cotton tensile strength loss (TSL)

**Fig. 3:**
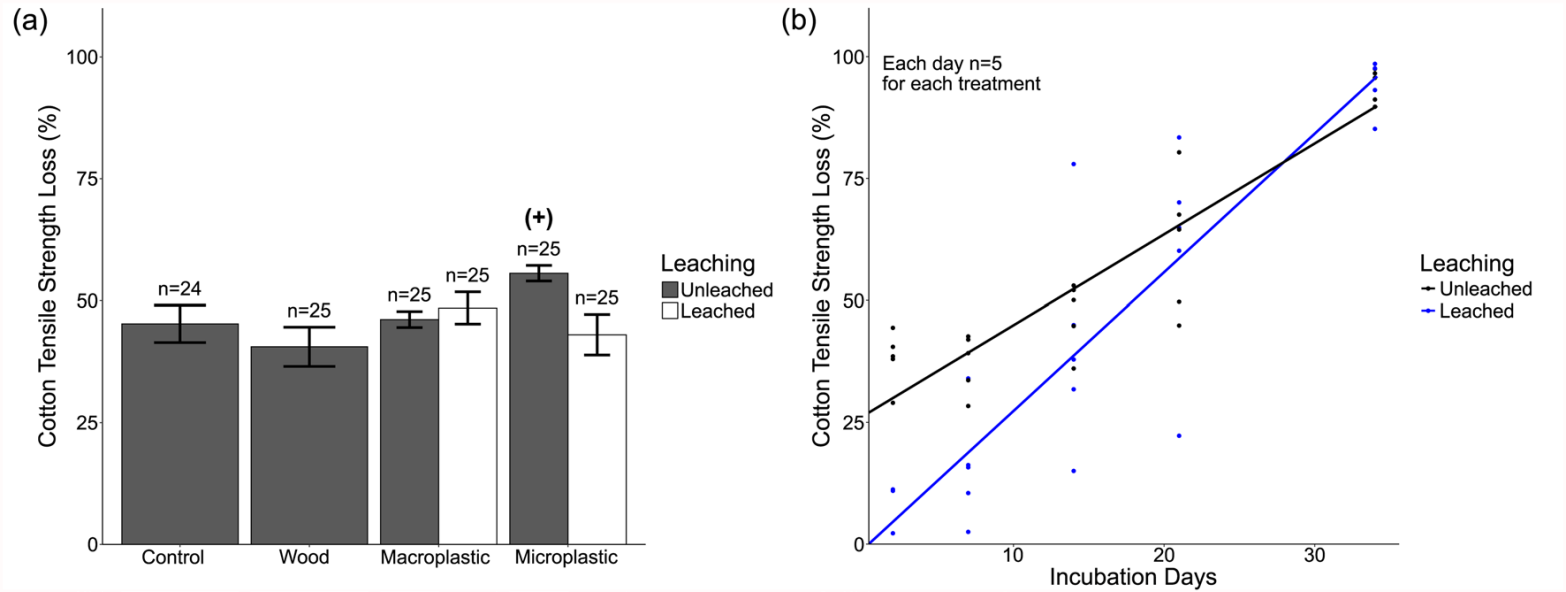
(**a**) Mean (± SE) cotton tensile strength loss when exposed to wood or different plastic treatments, averaged across all sampling dates (2, 7, 14, 21 and 34 days). Statistically significant differences between material addition treatments and controls identified using log response rations (Supplementary Fig. S3b) are denoted with (+) and (-) to indicate positive or negative effect sizes respectively. (**b**) Cotton tensile strength loss over time when exposed to unleached or leached microplastics. Lines represent predicted trends based on the mixed model. Only the microplastic treatments are shown, with visualisations for other treatments shown in Supplementary Fig. S4.

Cotton TSL was significantly affected by material (F_2,133_ = 3.56, *p* = 0.006), with 7.3% higher TSL in the presence of plastics relative to controls (Supplementary Fig. S4a). Additionally, there was a significant interaction between particle size and leaching status nested within material (F_1,133_ = 18.2, *p* < 0.001), with TSL elevated under the unleached microplastics treatment compared with the leached microplastic and both the unleached and leached macroplastic treatments (Fig. 3a). Based on log response ratio analysis, TSL increased by 23.4% when exposed to unleached microplastics relative to controls (Fig. 3a, Supplementary S4b). The three-way interaction between incubation time, particle size and leaching, nested in material, was also significant (F_1,133_ = 12.4, *p* < 0.001; Supplementary Fig. S5). TSL was higher on the two earliest sampling days (day 2 and 7) when exposed to unleached microplastics relative to leached microplastics, but both treatments had comparable TSLs by the end of the experiment (Fig. 3b). TSL was significantly affected by incubation time (F_1,133_ = 469, *p* < 0.001), with 643% higher mean TSL on day 34 than day 2 of incubation in the field.

### Fungal community biomass

**Fig. 4:**
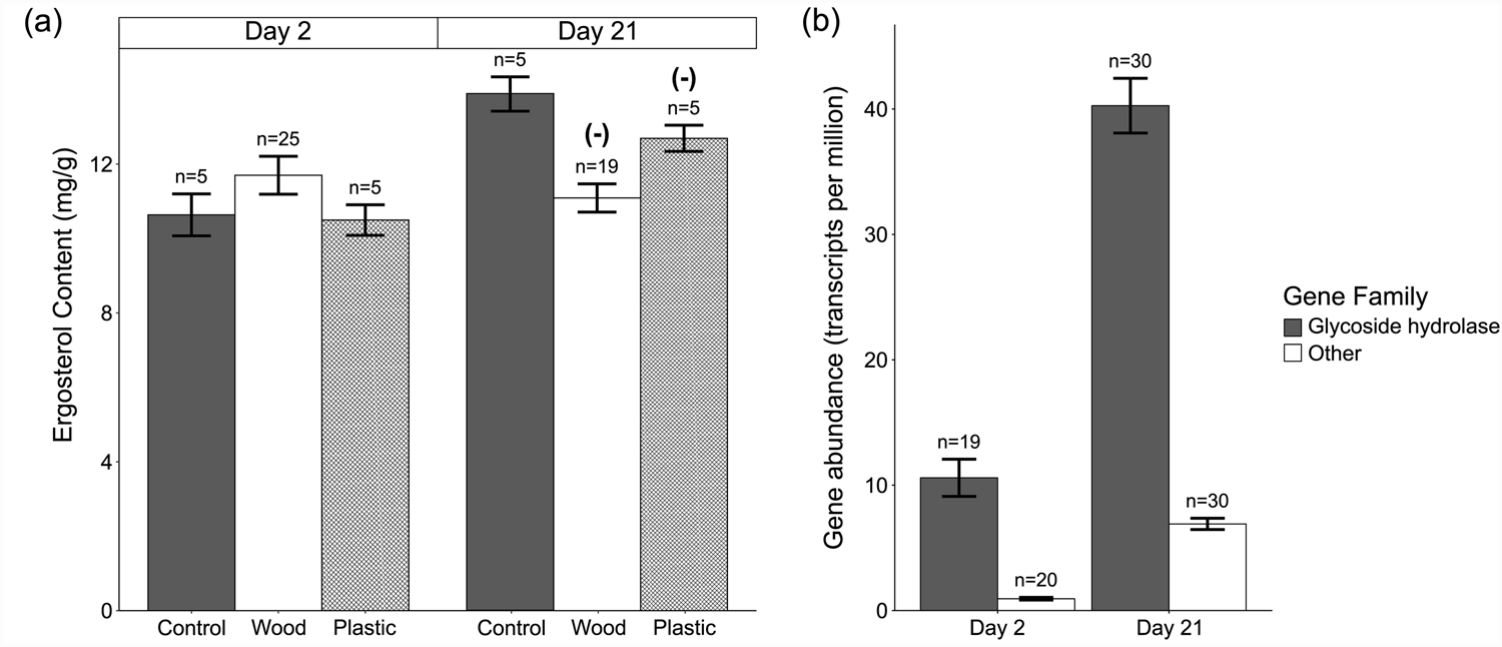
(**a**) Mean (± SE) fungal biomass associated with leaf litter when exposed to wood or plastic. Statistically significant differences between material addition treatments and controls identified using log response rations relative to the controls of each respective incubation day (Supplementary Fig. S5) are denoted with (+) and (-) to indicate positive or negative effect sizes respectively. (**b**) Mean (± SE) transcripts per million of functional genes associated with cellulose degradation on day 2 and day 21. Data is averaged across treatments, with visualisation for all treatments shown in Supplementary Fig. S6. Log response ratios could not be calculated due to the lack of control data on day 2.

Ergosterol, a primary component of fungal cell walls, was quantified as a proxy for fungal biomass^64^. Ergosterol content analysis of leaf litter showed an increase in mean fungal biomass of 14.0% over the incubation period (F_1,47 =_ 15.1, *p* < 0.001). Fungal biomass was also affected by an interaction between time and material (F_2,47 =_ 3.50, *p* = 0.038), with reduced fungal biomass on day 21 when exposed to wood and plastics relative to controls (Fig. 4a). On day 21, exposure to wood and plastic (averaged across size and leaching status) were associated with 20.1 and 8.6% lower mean biomass loss relative to the control treatment respectively.

### Fungal community composition

Of reads that could be assigned to the main fungal decomposer phyla, 86.7 ± 0.5% were Ascomycota and 11.3 ± 0.5% Basidiomycota. The dominant classes were Sordariomycetes (51.5 ± 1.0%), Saccharomycetes (13.9 ± 0.5%) and Eurotiomycetes (9.1 ± 0.1%). Dominant genera were Ascomycota: *Fusarium* (9.9 ± 0.2%), *Colletotrichum* (6.6 ± 0.1%) and *Aspergillus* (6.1 ± 0.1%).

**Fig. 5:**
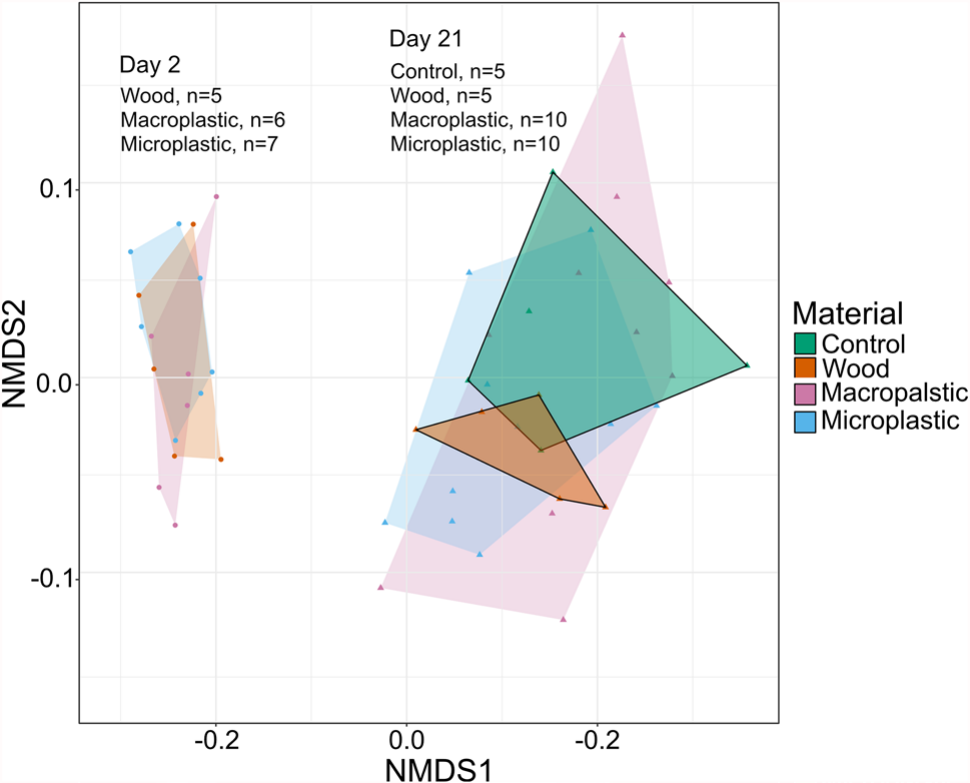
NMDS ordination of fungal communities with convex hulls overlaid for by material type and plastic size treatments. There is no control treatment data for day 2 due to insufficient DNA retrieval from samples for sequencing. Stress = 0.0725.

Fungal communities were clearly separated in the nMDS ordination between samples collected on day 2 and 21 (Fig. 5). This is supported statistically with a PERMANOVA, which showed a significant effect of sampling day on community composition (PERMANOVA, F_1,37 =_ 40.5, *p* = 0.001). Tests of multivariate variance indicated significant differences in dispersion between sampling days (PERMDISP *p* < 0.001), and among material (*p* = 0.010) and particle size (*p* = 0.018) treatments. Due to the strong separation of communities between the two sampling days, separate PERMANOVA tests were conducted for day 2 and day 21 communities, for which the assumption of homogenous multivariate variance was met in both analyses (both (PERMDISP *p* > 0.100). On day 2, fungal community composition did not vary among particle addition treatments (PERMANOVA all *p* > 0.050). On Day 21, however, community composition was significantly influenced by particle size nested in material (PERMANOVA F_1,24_ = 2.35, *p* = 0.045). Based on the nMDS plot, data points for litter packs exposed to both macroplastics and microplastics on Day 21 were more dispersed relative to microplastics, reflecting greater variability and reduced consistency in fungal community composition among replicates (Fig. 5, outlined hulls). In contrast, the data points for control and wood-treated litter packs on Day 21 were relatively compact and showed minimal overlap, indicating lower within-group variation and suggesting that wood shavings induced a distinct shift in community composition relative to controls (Fig. 5, although proportions of the 10 most important genera appeared broadly similar, visualized in Supplementary Fig. S7-S8).

### Fungal gene abundance: cellulose degradation

There were no significant effects of material treatments on the abundance of genes associated with cellulose degradation (all *p* > 0.050). There were significant effects of time (F_1,73 =_ 396, *p* < 0.001) and gene family (F_1,72 =_ 273, *p* < 0.001). Overall gene abundances increased by 318% between day 2 and day 21, and glycoside hydrolase family genes were 537% more abundant than the other gene families (Fig. 4b). Additionally, there was a significant interaction between time and gene family (F_1,72 =_ 10.3, *p* = 0.002). However, log response ratios could not be calculated to quantify the precise changes because control data were not available for day 2.

### Medical mask and wood shaving leachates

The main leachates of FMs were lauryl diethanolamide, erucamide, oleamide, 3-(Octyloxy)propan-1-amine and 1,3-di-o-Tolylguanidine. Compounds in wood leachate were identified to be 47.7% carboxyl-rich alicyclic molecules (also known as lignins), 23.3% carbohydrates, 14.1% tannins, 9.5% lipids, 5.0% unsaturated hydrocarbons and 0.4% condensed aromatics (Supplementary Fig. S10).

## Discussion

In this pond experiment, the presence of wood shavings and FM-derived PP plastics, refractory particles derived from natural and anthropogenic materials respectively, had distinct effects on leaf litter and cotton cellulose decomposition, and on fungal communities. Both wood shavings and plastics suppressed fungal biomass, but the presence of wood caused an approximately twofold greater reduction compared to plastics. The hypothesis of reduced leaf litter decomposition under exposure to refractory particles was only partially supported. As predicted, exposure to wood led to a reduction (~ 4%) in leaf litter decomposition, whereas exposure to macroplastics was associated with a very small (0.3%) increase. In contrast, cotton tensile strength loss increased by 6% in the presence of plastics overall, and by 22% in the presence of unleached microplastics, whereas wood had no effect. These findings illustrate that both natural (wood) and anthropogenic (plastic) refractory materials can suppress fungal biomass in alder leaf litter, an example of a relatively labile, naturally occurring, detrital substrate^65^, and alter detrital decomposition. However, plastic effects often contrasted with those of woody detritus, were generally smaller in effect size, and varied by particle size and leaching status.

### Effects of plastics

Fungal decomposers are the primary agents driving microbial hydrolysis of leaf litter in freshwaters^66,67^ through their ability to produce a diverse suite of enzymes to degrade the different structural polymers present in plant material^30^. Fungal biomass was reduced in the presence of plastic particles, which may act as a physical barrier limiting the spread of fungal hyphae. Plastic leachate compounds were instead characterized by common plastic additive compounds used to modify material properties^68^, such as surfactants and slip agents. Although some of these chemicals may contribute towards microbial growth inhibition, e.g. oelamide and erucamide slip agents can reduce biofouling on surfaces^69^, they are not explicitly antimicrobial, and are likely to have weaker effects relative to the antimicrobial compounds leaching from e.g. wood. The macroplastics treatment was hypothesised to have a stronger barrier effect, but it appears that the difference in size and degree of dispersion of plastic particles in the litter bags did not significantly influence fungal community biomass or composition. This suggests that the presence of plastics per se, rather than particle size, and their material properties (e.g. surface characteristics) may be driving effects on fungal communities. Fungal communities exposed to plastics showed broader compositional variation in nMDS ordination space compared with those in both wood shaving and control treatments, overlapping with both. We thus hypothesize that the novelty of plastics as a substrate does not favour development of specialist fungal communities such as those associated with wood, but rather contribute towards the development of broad generalist communities, such as those observed in synthetic industrial systems^70,71^.

Despite reduction in fungal community biomass, there was no significant corresponding decreases in leaf litter mass loss when exposed to plastics. In contrast to the hypotheses, we observed that decomposition of our cellulose (cotton) strips – a structurally simpler substrate than leaf litter – was stimulated in the presence of plastics overall, with the strongest effects observed during the early stages of the experiment for unleached microplastics. This suggests that the rapid release of leachates from microplastic particles might enhance the initial decomposition of simpler carbon substrates, but that these effects diminish over time as the leachates dissipate. A potential explanation for this is a shift in the dynamics of fungi and bacteria, where bacteria productivity is increased while those of fungi is reduced, similar to dynamics observed under elevated nutrient availability^72^. In this study, plastic leachates may represent an additional nutrient source for microorganisms. Plastic leachates contain plastic-derived dissolved organic carbon, which can be more bioavailable than some forms of natural dissolved organic matter and stimulate microbial growth^73–75^. Further, the plastic leachates in this study contained lauric diethanolamide, a compound previously shown to enhance microbial biodegradation of mineral hydraulic oil by potentially supplying nitrogen that supports microbial growth^76^. As the pond used in this experiment has low concentrations of inorganic nitrogen (< 0.1 mg L^− 1^; Supplementary Table S1), such nitrogen supplying compounds may stimulate bacterial activity.

In contrast with the stimulatory effect of unleached microplastics on the earliest stages of cellulose decomposition, macroplastics did not have significant effects on overall litter decomposition. Microplastics likely leach compounds more quickly than macroplastics due to their higher surface-area-to-volume ratio^77,78^, potentially resulting in a stronger, immediate response from microbial activity and decomposition processes that diminishes over time as the leachates from plastics are exhausted. The microplastic particles in this study were estimated to have a total surface area of 163 cm^2^ compared to the 146 cm^2^ of macroplastics. In the environment, larger plastic waste items, such as facemasks, would likely release easily leachable chemicals prior to fragmentation into microplastics. However, microplastics can still leach out chemicals such as dissolved organic matter across longer time periods, albeit at lower rates^79^. These results highlight the importance of further research into the prolonged ecological effects of plastic leachates on microbial communities and ecosystem functioning.

### Effects of wood shavings

The presence of wood shavings had a stronger negative effect on fungal biomass than plastics, compared with particle free controls, and unlike plastics reduced litter decomposition. The observed suppression of fungal biomass may be linked to the presence of naturally-occurring inhibitory polyphenolic compounds leached from wood shavings, including lignins, and tannins – compound classes known to inhibit not just fungal, but overall microbial activity^80,81^. In addition to chemical inhibition, wood shavings might also present a physical barrier that inhibits the spread of fungal conidia and hyphae through more labile detritus in the litter pack, limiting fungal growth^82^. Leaf litter fungal communities exposed to wood shavings were also compositionally distinct from those in the controls by the end of the experimental period. This shift may result from the same chemical and physical effects of wood shaving exposure that suppressed fungal biomass. Additionally, competitive interactions with wood-associated fungi, which are known to inhibit the growth and activity of leaf-litter-associated fungi through production of antifungal compounds^83^, may further shape the structure of fungal communities in the broader litter pack.

### Implications

Freshwater ecosystems, specifically those close to urban areas such as the pond investigated here, are particularly vulnerable to pollution from a wide range of anthropogenic waste products. Among these, disposable FMs are of particular concern, as they are typically discarded intact after a short period of use, and may regularly reach freshwater habitats with minimal leaching. Accordingly, the effects of FM derived plastics observed in this investigation warrant attention, given FM use among the general public is widespread in some regions of the world and is likely to remain a key component of coordinated responses to future respiratory disease outbreaks^8,9^. More generally, the results documented here demonstrate the potential for plastic particles to affect freshwater fungi and the ecosystem processes they regulate.

The effects observed here, of both plastic and wood presence on freshwater fungi and detritus decomposition, are modest compared to larger effect sizes reported for other environmental stressors, including salinity^84^, wastewater^85^, nutrient enrichment^86^ and warming^87^. Nevertheless, even subtle shifts in key ecosystem processes can have broader ecological consequences. For example, increased decomposition of organic matter could alter carbon storage in freshwater wetlands, potentially affecting their role as carbon sinks^88^. Given the increasing prevalence of macro- and microplastic pollution in freshwaters, further research is needed to understand how the changes in fungi communities and detritus decomposition documented here might influence freshwater food webs and ecosystem integrity over time.

## Materials and methods

### Study site

The field study was conducted from May to July 2022 in an ornamental pond in the “Kunskapsparken” botanical garden, located at the Swedish University of Agricultural Sciences, Uppsala (59.816361 N, 17.664576E). The pond is a naturally formed “kettle hole” with a thick layer of benthic organic sediment (between 20 and 45 cm) and a maximum water depth of 1.7 m. Due to the managed nature of the surrounding garden, the diversity and amount of leaf litter input into the pond is likely to be different from unmanaged ponds.

### Experimental design and treatments

The litter bags were assigned to one of six treatments to assess the effects of added materials (plastic or wood) on decomposition. The treatments consisted of alder leaves mixed with (1) wood shavings, (2) unleached PP macroplastic, (3) leached PP macroplastic, (4) unleached PP microplastic, or (5) leached PP microplastic. A sixth treatment served as a control with no added material apart from leaf litter. After treatment application, litterbags were sealed with cotton thread. Leaf litter mass loss was measured after 2, 7, 14, 21, and 34 days.

Wood shavings consisted of untreated dust-free conifer shavings sold as bedding for pet animals (Dammfritt Burspån, Dogman) and had a diameter range of 0.2–1.4 mm (mean = 0.6 mm). Wood shavings were sieved through a 0.5 mm mesh to ensure that only particles larger than the cotton bag mesh size (0.5 mm) were used. Although the wood shavings were not produced through a natural process, they serve as a surrogate for naturally occurring, highly refractory, organic matter in this study.

Plastic particles were derived from type IIR medical masks (EN 14683:2019, Verdent sp. z o.o. Poland), specifically the blue melt-blown PP fibre sheets. To prepare the leached plastic particles, five PP sheets were submerged in 800 ml MilliQ^®^ ultrapure water and left to leach for 48 h at room temperature with occasional stirring^89^. Both leached and unleached PP sheets were then air-dried for 24 h, after which macro- (60 × 60 mm) and micro- (3 × 3 mm) plastic particles were prepared by cutting the PP sheets with scalpel and scissors. Previous research has shown that the concentrations of plastic leachates peak at approximately 12 h when leached in deionized water^89^. A 48-hour leaching period was applied to PP sheets to remove most of the readily leachable plastic compounds. PP sheets were then cut into appropriate sizes.

### Preparation of litter bags

Cotton tea bags (100 × 150 mm, mesh size: 0.5 mm, Belle Vous) were used as litterbags, rather than the plastic mesh bags typically used in leaf litter decomposition assays, to minimize additional plastic effects on the litter packs. Each bag was filled with 3.5 g of dried alder leaves, which were collected as freshly abscised leaves in the autumn of 2017 from an alder forest stand near Ultuna, Uppsala (59.811177 N, 17.669750E) that is not subjected to anthropogenic fertilization.

For treatments with the addition of particles, 0.2 g of either plastic or wood shavings were added into the bag prior to addition of the leaf litter. For macroplastics, two PP sheets were placed on opposite inner sides of the bag, with leaf litter subsequently inserted in between. Wood shavings and microplastics were introduced by vigorously rubbing the sides of the bag together to disperse the particles throughout. Leaf litter was then added, and the entire bag shaken to ensure random distribution. For microplastic and wood shavings, the weight added corresponds to approximately 400 and 215 particles respectively. During the preparation process, only metal, glass, or wooden tools were used, and cotton clothing was worn to minimize plastic contamination. All surfaces were regularly cleaned with 70% ethanol and overhead extraction fans were used to reduce airborne microplastic contamination.

### Decomposition assays

The litterbags were incubated for 5 weeks, with 5 sampling dates and one replicate of each treatment randomly allocated to one of five experimental blocks (Fig. 1). Litterbags were evenly spaced and fastened to links of stainless-steel chains and then sunk onto the benthic substrate. We recorded the water temperature every 30 min using temperature loggers (SmartButton, ACR Systems Inc., Surrey) attached to each chain (Supplementary Fig. S9). The litterbags were deployed in near-shore areas of the pond on the 31 st of May 2022 and retrieved after 2, 7, 14, 21 and 34 days.

### Measurements

#### Leaf mass loss

Litterbags were retrieved from the field on each of the five collection dates (Fig. 1). Each litterbag and its contents were dried separately at 50 °C for 48 h. After drying, plastic particles or wood shavings were carefully separated from leaf litter with tweezers. The litter remaining was weighed to determine mass loss as a measure of decomposition over the incubation period.

#### Tensile strength loss (TSL)

In this study, TSL was used as a measure of freshwater cotton cellulose decomposition, when associated with leaf litter. One cotton strip (80 × 25 mm) was cut from each dried litterbag and tested for tensile strength using a motorized force tester (Mark-10; Motorized Tension/Compression Test Stand ESM303) at a constant speed of 20 mm/min. Peak tension was measured with a digital force gauge (Mark-10; Series 5, Force gauge model M5-100). TSL was calculated as the difference between the measured peak tension and a baseline value. The baseline value was estimated based on the average peak tension of 30 new cotton bags, never exposed in the field.

### Fungal community biomass, composition and functional gene abundance

For each litterbag retrieved on day 2 and 21, five leaves were randomly selected and three 6 mm discs cut from each. One disc from each of the five leaves selected from a litterbag was pooled together in a set, resulting in three sets per litterbag, each containing five discs. The first set was dried and weighed as the rest of the leaf litter and used to estimate the total mass remove from leaf litter by disc sampling. The remaining two sets were stored at −20 °C, and later used for ergosterol content analysis and DNA extraction, respectively.

#### Ergosterol content analysis

Leaf discs were freeze dried and analysed at the University of Applied Sciences and Arts of Southern Switzerland (SUPSI). Solid-phase extraction (Sep-Pak^®^ Vac RC tC18 500 mg sorbent; Waters, Milford, USA) was used to extract and purify ergosterol^90^, which was then quantified using ultra-high-performance liquid chromatography (UHPLC; 1250 Infinity Series, Agilent Technologies, Santa Clara, USA) at 282 nm and a column temperature of 33 °C.

#### High throughput sequencing

Total genomic DNA was extracted from one set of leaf discs from each litterbag using DNeasy PowerSoil kits (Qiagen), following the manufacturer’s protocol. The quality and quantity of the extracted DNA were assessed using a Qubit fluorometer (Invitrogen). We used a combination of sample-specific short read sequencing together with long read sequencing of a pooled sample combining aliquots from each individual short-read sample. The long reads serve as a reference to classify and annotate the short reads. Raw-reads from both sequencing approaches were provided as ‘fastq’ files for further bioinformatics analysis.

For short-read sequencing, 200 ng of DNA of each sample was provided to the SNP&SEQ Technology Platform at NGI-SciLifeLab (Uppsala University, Sweden). Libraries were prepared using the SMARTer ThruPLEX DNA-seq kit (Takara Bio) and sequenced through 150 cycles paired-end reads on one lane of a S4 flowcell using the NovaSeq 6000 platform (v1.5 sequencing chemistry, Illumina Inc.), generating ~ 10 million reads per sample.

For long-read sequencing, DNA samples were pooled into a 2000 ng meta-sample and sequenced at the Uppsala Genome Centre (UGC) at NGI-SciLifeLab (Uppsala University, Sweden) using the Oxford Nanopore PromethION Technology Platform. DNA sample quality and quantity is performed prior to library construction using Qubit Fluorometric Quantitation (Thermo Fisher Scientific) and AgilentBioanalyzertechnologies. Samples were purified using Ampure beads prior to library preparation.

Fungal DNA extraction and sequencing was successful for all samples except control litterbags on day 2, where DNA yields were extremely low.

### Bioinformatic analysis

Metagenomic raw data (fastq files) were analysed using nf-core/mag and nf-core/metatdenovo pipelines of the nf-core collection of workflows utilising reproducible software environments (for details refer https://nf-co.re/mag and https://nf-co.re/metatdenovo/dev/, both accessed on 20 July 2023). In brief, raw sequencing reads (from short read illumina sequencing) were quality filtered using fastp (v0.39) to remove sequencing adapters and low-quality bases, followed by removal of host reads using bowtie2 (v2.4.2) against the alder reference genome (ncbi accession: GCA_003254965.1). Taxonomic profiling of the high-quality reads was done using Kraken2 classifier^91^(v2.1.2) against a fungal reference database containing taxa primarily from the Ascomycota and Basidiomycota phyla (RefSeq, accessed on September 2023). The taxonomic classification results were used for downstream compositional analysis. Functional groups were identified using taxa lists referenced against Fungal traits database^92^. We managed to assign ~ 1.0 ± 0.1% of reads to fungi, similar to what is obtained for the hypolimnion of lakes^93^.

Quality long reads (from the nanopore sequencing) were assembled using *de novo* flye assembler (v 2.9.1-b1780) with *meta* option enabled. To identify putative protein-coding regions, assembled contigs (continuous sequences generated from overlapping reads) were identified to Open Reading Frames (ORFs) using prediction tool prodigal (v 2.6.3). Cleaned short reads (illumina samples reads) were aligned back to the assembled contigs using BBMap (v39.01). Gene-level (read counts per ORF and sample) quantification was performed using featureCounts (v2.0.1). Functional annotation was conducted using eggNOG-mapper (v 2.1.9), which assigns orthologous groups and functional categories (COG, GO, KEGG) based on precomputed orthology relationships. ORF calling to featureCounts steps were done under the nf-core/metatdenovo (dev) reproducible pipeline. Genes related to cellulose degradation were identified using COG descriptions: glycoside hydrolase, beta-glucosidase, galactosidase, pectate lyase, carbohydrate esterase.

### Chemical screening of leachate

High-resolution mass spectrometry was used to perform suspect screening for the identification of leachable compounds from FM-derived PP sheets and wood shavings. One hundred grams of each material was suspended in 200 mL of MilliQ^®^ ultrapure water for 48 h. Leachates were then concentrated through evaporation and analysed using a Vanquish Horizon UPLC system coupled to a high-resolution mass spectrometer (QExactive Focus; Thermo Fisher Scientific, Bremen). The column oven of the UPLC was set to 40 °C. Further details on sample and data analysis can be found in the supplementary information.

### Statistical analysis

We used linear mixed-effect models (LMMs) to test the main effects of particle *material* (three levels: control, wood shavings, plastics) and *time*, both fitted as fixed effects, with *litterbag chain* fitted as a random block effect to account for environmental heterogeneity (e.g. water depth, surrounding vegetation). Two further fixed factors were nested and fully crossed within the factor *material*: *leaching* (leached and unleached) and *particle size* (macro and microplastic). Time was modelled as a continuous variable for most responses, except for fungal community analyses, which were measured at only two time points and analysed accordingly. The base model is thus:

$$\:Response\:\sim\:time\:\times\:\frac{Material}{\left(Leaching\:\times\:particle\:size\right)}+\left(1\:\right|\:litterbag\:chain)$$

ANOVA tables for all univariate analyses are available in Supplementary Table S2. LMMs were fitted using the *lme4* R package^94^. The significance of fixed effects was tested through analysis of variance (ANOVA) using the *lmerTest* R package^95^, applying the Satterthwaite approximation to estimate F- and p-values. Response variables were log10-transformed where necessary to meet ANOVA model assumptions (Supplementary Table S2).

As a further test to evaluate variation among all treatments, including nested factors, relative to controls, log response ratios (LRRs) were calculated for all response variables by dividing each treatment value by its paired control value within the same block and log-transforming the result^21^. Treatment effects were considered significantly different from controls when 95% confidence intervals did not overlap zero.

Multivariate variation in fungal community composition was analysed using non-metric multidimensional scaling (nMDS) based on Bray–Curtis dissimilarities calculated from relative taxon abundances. Differences in fungal community composition among groups were then tested using permutational analysis of variance (PERMANOVA). PERMANOVA analyses included the same treatment effects as the univariate LMMs and accounted for the randomised block design by restricting permutations within *litterbag chain*. Homogeneity of multivariate dispersion was assessed using permutation tests of multivariate dispersions (PERMDISP) using R package *vegan*. Both nMDS and PERMANOVA analyses were implemented using the *vegan* R package^96^. PERMANOVA output tables are available in Supplementary Table S3.

Unless stated otherwise, all reported F statistic and p values in this paper are derived from ANOVAs. All plots were done using R package *ggplot2*^97^.

## Supplementary Information

Below is the link to the electronic supplementary material.

Supplementary Material 1

## Data Availability

Raw sequencing data is deposited in ENA repository with study accession PRJEB101937 for short read raw sequences ([https://www.ebi.ac.uk/ena/browser/view/PRJEB101937](https:/www.ebi.ac.uk/ena/browser/view/PRJEB101937)) and PRJEB101937 for long read raw sequences ([https://www.ebi.ac.uk/ena/browser/view/PRJEB101937](https:/www.ebi.ac.uk/ena/browser/view/PRJEB101937)). Other data generated and analysed during the current study are available from the corresponding author on reasonable request.

## Code availability

Detailed bioinformatics workflows or commands, scripts and parameters can be found in the GitHub repository: (https://github.com/abu85/shotgun_metagenomic_data_analysis).
