# Supplementary material for: Medical facemask waste alters detritus decomposition and fungal communities in a freshwater pond
Source: Sci Rep. 2026 Mar 30;16:10597. doi: 10.1038/s41598-026-45795-5 (PMC13039742; doi:10.1038/s41598-026-45795-5)
Supplement: Supplementary file 1 — Supplementary Material 1 [file 41598_2026_45795_MOESM1_ESM.docx]

**Supplementary Information for** *Medical facemask waste alters detritus decomposition and fungal communities in a freshwater pond – Kong et al. (2026)*


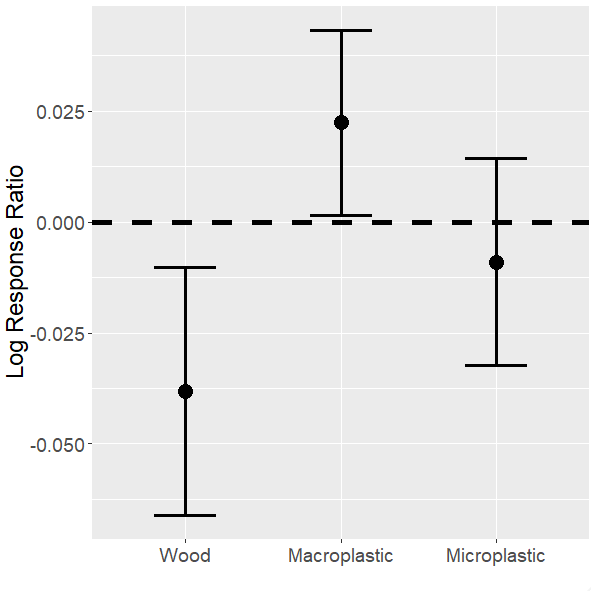


Figure S1. Log response ratio of leaf litter decomposition when exposed to particle of different materials. Error bars are the 95 % confidence interval.


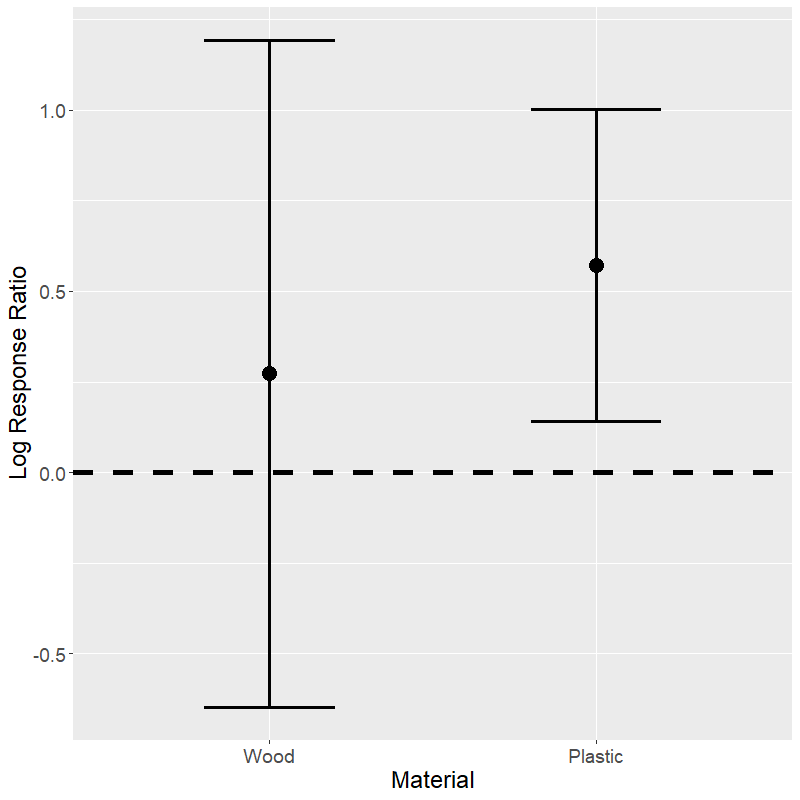

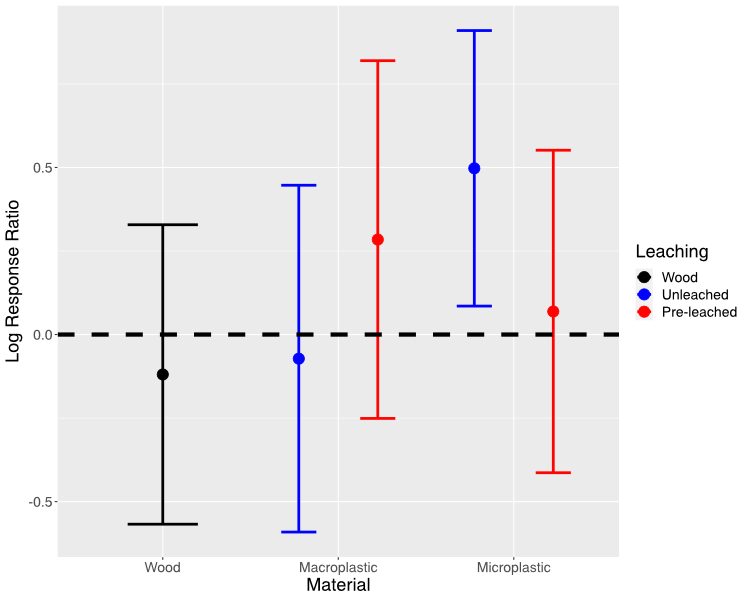


(a)

(b)

Figure S3. Log response ratios of (a) cotton tensile strength loss when exposed to wood or plastic, and (b) cotton tensile strength when exposed to different plastic particle sizes and pre-leaching treatments. Error bars are the 95 % confidence interval.


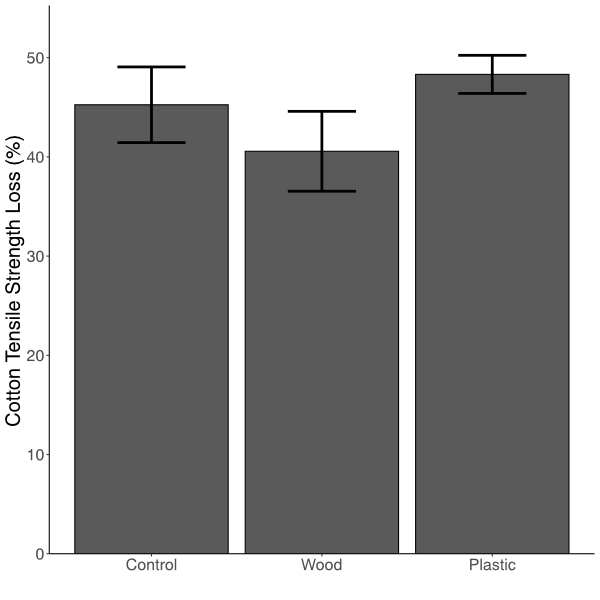


Figure S2. Mean (± SE) leaf mass loss when exposed to wood or either macro- or microplastics, averaged across all sampling dates (2, 7, 14, 21 and 34 days). Statistically significant treatments based on analysis of log response rations (Supplementary file Fig. S5a) are denoted with (+) and (-) to indicate positive or negative effect sizes respectively.

Figure S4. Cotton tensile strength loss over time when exposed to particles of different materials and pre-leaching treatments. The grey area around the regressions indicate the 95 % confidence interval.


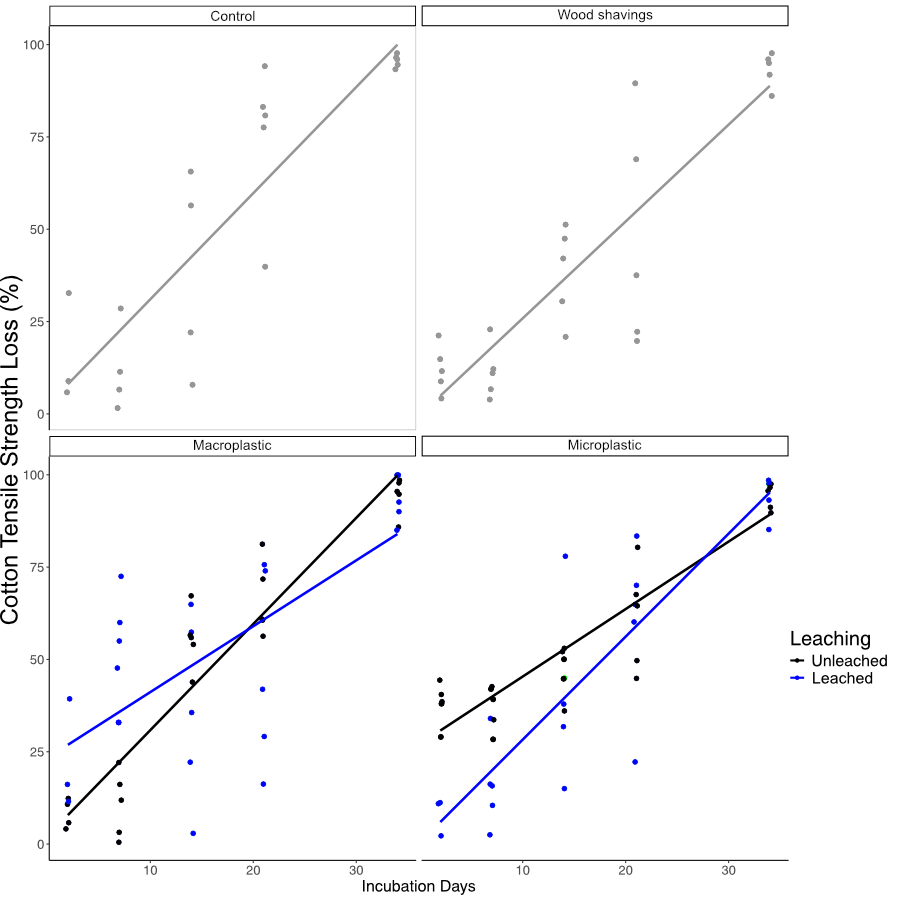

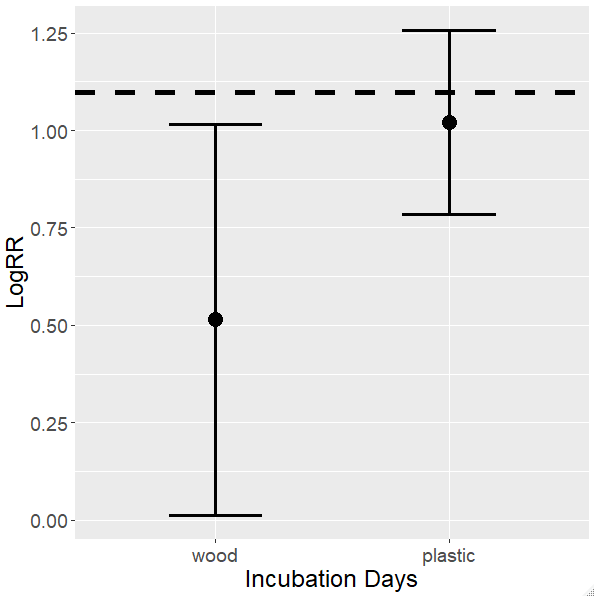


Figure S5. Log response ratios of ergosterol content change between day 2 and 21 of the experiment when exposed to particles of different materials. Error bars are the 95 % confidence interval.


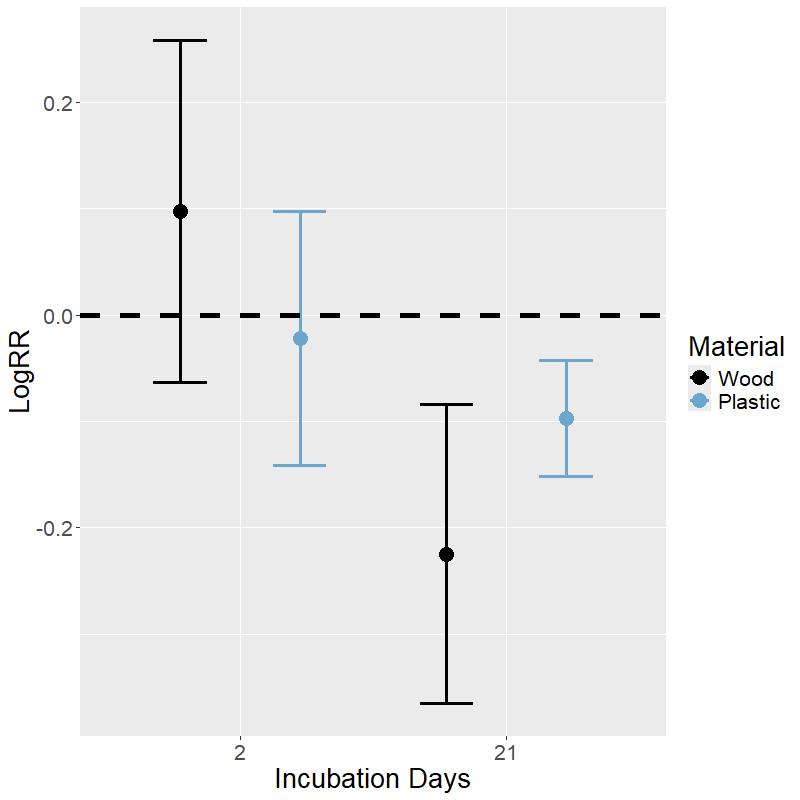


*
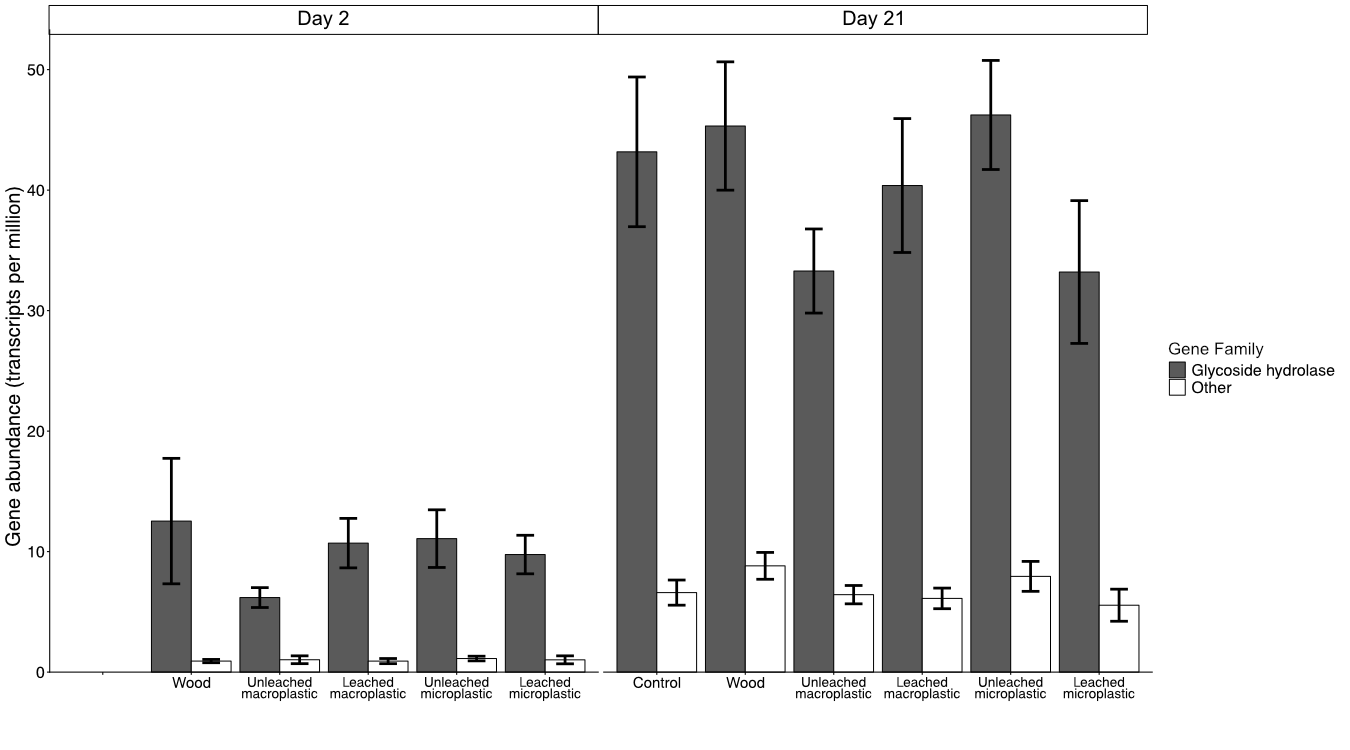
*

Figure S6 - Mean (± SE) transcripts per million of functional genes associated with cellulose degradation on day 2 and day 21. The lack of data for the Control treatment on day 2 is due to insufficient DNA concentration from the collected material.


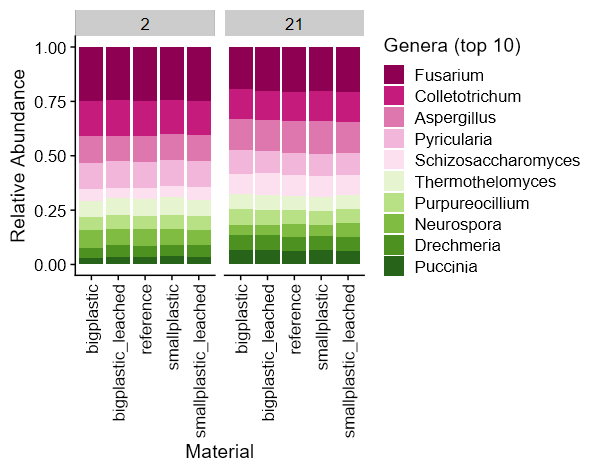


Unleached Macroplastic

Pre-leached Macroplastic

Wood

Unleached Microplastic

Pre-leached Microplastic

Unleached Macroplastic

Pre-leached Macroplastic

Wood

Unleached Microplastic

Pre-leached Microplastic

Figure S7. Top 10 genera of fungi identified across different plastic exposure treatments and sampling day.

Figure S8. Relative abundances of fungal lifestyle strategies across different plastic exposure treatments and sampling day.


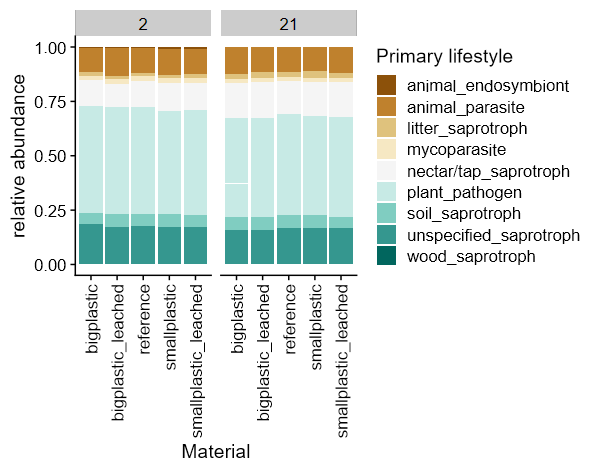


Unleached Macroplastic

Pre-leached Macroplastic

Wood

Unleached Microplastic

Pre-leached Microplastic

Unleached Macroplastic

Pre-leached Macroplastic

Wood

Unleached Microplastic

Pre-leached Microplastic

Table S1. Pond water values for total nitrogen, total phosphorus, dissolved organic carbon, nitrite + nitrate nitrogen, phosphate phosphorus and ammonium nitrogen for two sample dates before and during the experiment period. Data is provided by Michael Peacock via the LEAF-PAD project, with sample processed by the SLU Geochemical laboratory. Pond water was collected from the shore edge just below the water surface.

| Sampling date | Tot-N_TNb  μg/l | Tot._P  μg/l | DOC  mg/l | NO2+NO3_N  μg/l | PO4_P  μg/l | NH4_N  μg/l |
| --- | --- | --- | --- | --- | --- | --- |
| 19-May-22 | 788 | 183 | 11.3 | <1 | 19 | 7 |
| 15-Jun-22 | 1020 | 134 | 11.1 | 1 | 48 | 26 |


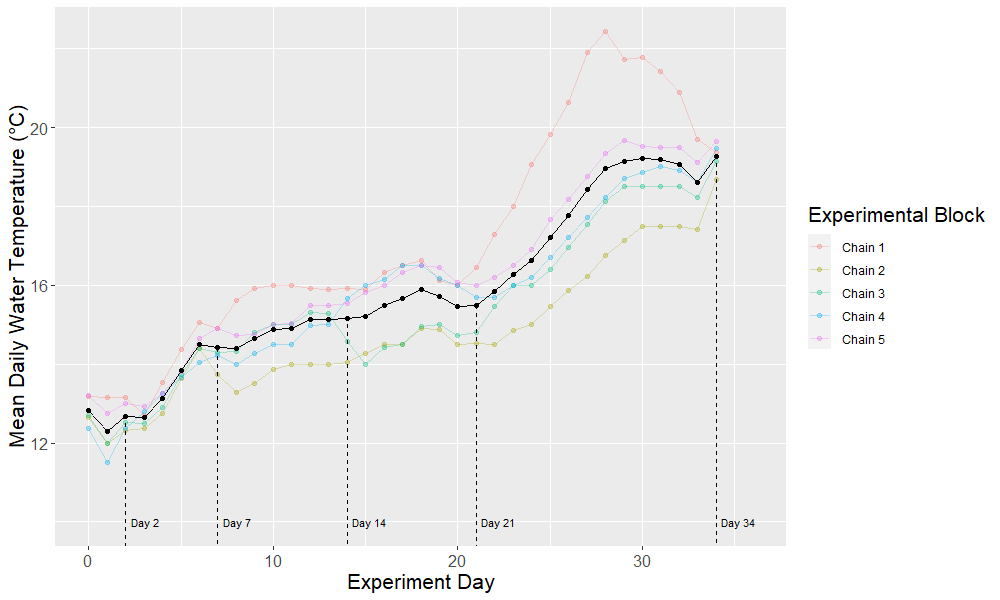


Figure S9. Changes in daily average pond temperature over the course of the experiment in May and June of 2022, recorded by temperature loggers attached to each chain (experimental block). The black line indicates the mean temperatures averaged across all chains. Vertical lines indicate when decomposition bags were removed from the pond. Temperature loggers were set to record temperatures every 30 mins.

Table S2. ANOVA output table of linear mixed models testing the effects of treatments and time on measured responses.

|  | Sum Sq. | Mean Sq. | NumDF | DenDF | F-value | Pr(>F) |
| --- | --- | --- | --- | --- | --- | --- |
| **Leaf litter decomposition (%) – log10 transformed** | | | | | | |
| Incubation.day | 2.109 | 2.109 | 1 | 133 | 2300 | **<0.001** |
| Material | 0.003 | 0.002 | 2 | 133 | 1.78 | 0.172 |
| Material(Size) | 0.005 | 0.005 | 1 | 133 | 5.89 | **0.017** |
| Material(Leaching) | 0.000 | 0.000 | 1 | 133 | 0.12 | 0.731 |
| Material(Size*Leaching) | 0.002 | 0.001 | 2 | 133 | 1.29 | 0.278 |
| Incubation.day*Material | 0.000 | 0.000 | 1 | 133 | 0.05 | 0.825 |
| Incubation.Day*Material(Size) | 0.002 | 0.002 | 1 | 133 | 1.89 | 0.172 |
| Incubation.Day*Material(Leaching) | 0.000 | 0.000 | 1 | 133 | 0.10 | 0.748 |
| Incubation.Day*Material(Size*Leaching) | 0.000 | 0.000 | 1 | 133 | 0.02 | 0.887 |
|  |  |  |  |  |  |  |
| **Cotton tensile strength loss (%)** | | | | | | |
| Incubation.day | 9.503 | 9.503 | 1 | 125 | 415 | **<0.001** |
| Material | 0.241 | 0.120 | 2 | 125 | 5.25 | **0.006** |
| Material(Size) | 0.001 | 0.001 | 1 | 125 | 0.03 | 0.864 |
| Material(Leaching) | 0.006 | 0.006 | 1 | 125 | 0.24 | 0.624 |
| Material(Size*Leaching) | 0.178 | 0.089 | 2 | 125 | 3.88 | **0.023** |
| Incubation.day*Material | 0.424 | 0.424 | 1 | 125 | 18.5 | **<0.001** |
| Incubation.Day*Material(Size) | 0.000 | 0.000 | 1 | 125 | 0.01 | 0.943 |
| Incubation.Day*Material(Leaching) | 0.001 | 0.001 | 1 | 125 | 0.05 | 0.819 |
| Incubation.Day*Material(Size*Leaching) | 0.304 | 0.304 | 1 | 125 | 13.3 | **<0.001** |
|  |  |  |  |  |  |  |
| **Leaf litter ergosterol content (mg/g) – log10 transformed** | | | | | | |
| Incubation.day | 0.285 | 0.285 | 1 | 47 | 15.1 | **<0.001** |
| Material | 0.021 | 0.010 | 2 | 47 | 0.55 | 0.583 |
| Material(Size) | 0.055 | 0.055 | 1 | 47 | 2.90 | 0.095 |
| Material(Leaching) | 0.001 | 0.001 | 1 | 47 | 0.05 | 0.821 |
| Material(Size*Leaching) | 0.132 | 0.066 | 2 | 47 | 3.50 | **0.038** |
| Incubation.day*Material | 0.003 | 0.003 | 1 | 47 | 0.16 | 0.688 |
| Incubation.Day*Material(Size) | 0.022 | 0.022 | 1 | 47 | 1.16 | 0.287 |
| Incubation.Day*Material(Leaching) | 0.018 | 0.018 | 1 | 47 | 0.93 | 0.339 |
| Incubation.Day*Material(Size*Leaching) | 0.012 | 0.012 | 1 | 47 | 0.62 | 0.434 |
|  |  |  |  |  |  |  |
|  | | | | | | |
| **Fungal community functional gene abundance – log10 transformed** | | | | | | |
| Day | 10.485 | 10.485 | 1 | 73 | 396 | **< 0.001** |
| Gene.Family | 7.247 | 7.247 | 1 | 72 | 273 | **< 0.001** |
| Material | 0.037 | 0.019 | 2 | 73 | 0.70 | 0.500 |
| Day*Gene.Family | 0.272 | 0.272 | 1 | 72 | 10.3 | **0.002** |
| Material(Size ) | 0.004 | 0.004 | 1 | 73 | 0.16 | 0.689 |
| Material(Leaching) | 0.001 | 0.001 | 1 | 73 | 0.04 | 0.849 |
| Day*Material | 0.025 | 0.025 | 1 | 73 | 0.95 | 0.333 |
| Gene.Family*Material | 0.053 | 0.027 | 2 | 72 | 1.00 | 0.371 |
| Material(Size*Leaching) | 0.004 | 0.004 | 1 | 73 | 0.16 | 0.686 |
| Day*Material(Size) | 0.000 | 0.000 | 1 | 73 | 0.01 | 0.907 |
| Day*Material(Leaching) | 0.010 | 0.010 | 1 | 73 | 0.39 | 0.533 |
| Gene.Family*Material(Size) | 0.005 | 0.005 | 1 | 72 | 0.19 | 0.666 |
| Gene.Family*Material(Leaching) | 0.041 | 0.041 | 1 | 72 | 1.53 | 0.220 |
| Day*Gene.Family*Material | 0.026 | 0.026 | 1 | 72 | 0.99 | 0.322 |
| Day*Material(Size*Leaching) | 0.014 | 0.014 | 1 | 72 | 0.52 | 0.474 |
| Gene.Family*Material(Size*Leaching) | 0.018 | 0.018 | 1 | 72 | 0.69 | 0.408 |
| Day*Gene.Family*Material(Size) | 0.002 | 0.002 | 1 | 72 | 0.07 | 0.788 |
| Day*Gene.Family*Material(Leaching) | 0.011 | 0.011 | 1 | 72 | 0.43 | 0.515 |
| Day*Gene.Family*Material(Size*Leaching) | 0.004 | 0.004 | 1 | 72 | 0.17 | 0.685 |
|  |  |  |  |  |  |  |

Table S3 – PERMANOVA output table testing the effects of treatments and time on microbial community composition based on Bray-Curtis dissimilarities.

|  | Df | SumOfSqs | R2 | F | Pr(>F) |
| --- | --- | --- | --- | --- | --- |
| **All data** | | | | | |
| Day | 1 | 0.255 | 0.474 | 40.50 | **0.001** |
| Material | 2 | 0.011 | 0.021 | 0.88 | 0.489 |
| Day*Material | 1 | 0.006 | 0.011 | 0.92 | 0.413 |
| Day*Material(Size) | 2 | 0.017 | 0.032 | 1.35 | 0.212 |
| Day*Material(Leaching) | 2 | 0.007 | 0.012 | 0.53 | 0.803 |
| Day*Material(Size*Leaching) | 2 | 0.010 | 0.018 | 0.79 | 0.550 |
| Residual | 37 | 0.233 | 0.433 |  |  |
| Total | 47 | 0.538 | 1.000 |  |  |
|  |  |  |  |  |  |
| **Day 2 Only** | | | | | |
| Material | 1 | 0.001 | 0.011 | 0.18 | 0.991 |
| Material(Size) | 1 | 0.006 | 0.078 | 1.24 | 0.303 |
| Material(Leaching) | 1 | 0.001 | 0.016 | 0.26 | 0.973 |
| Material(Size*Leaching) | 1 | 0.007 | 0.082 | 1.31 | 0.337 |
| Residual | 13 | 0.066 | 0.813 |  |  |
| Total | 17 | 0.081 | 1.000 |  |  |
|  |  |  |  |  |  |
| **Day 21 Only** | | | | | |
| Material | 2 | 0.014 | 0.048 | 0.69 | 0.547 |
| Material(Size) | 1 | 0.024 | 0.081 | 2.35 | **0.045** |
| Material(Leaching) | 1 | 0.005 | 0.018 | 0.52 | 0.664 |
| Material(Size*Leaching) | 1 | 0.008 | 0.027 | 0.79 | 0.411 |
| Residual | 24 | 0.246 | 0.826 |  |  |
| Total | 29 | 0.298 | 1.000 |  |  |
|  |  |  |  |  |  |

**Chemical screening of leachate: Plastic and Wood leachate Analyses**

The following is the procedure used to analyse the leachate of plastic and wood used in this experiment. The materials used for this analysis were separate from those used in the field experiment.

Sample preparation

Roughly 100 g of wood chips were suspended in 200 mL of ultrapure water (Milli-Q) for 48 h, after which it was aliquoted into a 50 mL falcon tube. The samples were dried under a gentle stream of nitrogen until dryness. The tubes were then frozen until time of analysis. When they were thawed, 1 mL of ultrapure water (Milli-Q) was added, the tubes were then vortexed for 1 minute and then ultrasonicated for 5 minutes to dissolve all dried material.

The facemasks were opened up with scissors where the white interior material was separated from the blue exterior material. The blue exterior sheet was suspended in 200 mL of ultrapure water (Milli-Q) for 48 hours, after which it was aliquoted into a 50 mL falcon tube. The tube was put under a gentle stream of nitrogen until 1 mL of water remained. The solution was the vortexed for 1 minute and ultrasonicated for 5 minutes to ensure that solid residue on the walls of the falcon tube was re-dissolved.

Instrumental analysis

The separation was performed using a Vanquish Horizon UPLC system coupled to a high-resolution mass spectrometer (QExactive Focus; Thermo Fisher Scientific, Bremen). The chromatographic analytical column used was a Waters Acquity UPLC C18 1.7 µm (2.1 x 50 mm), with a mobile phase of ultrapure water (Milli-Q) with 0.1 % formic acid and methanol (Merck, LC-MS grade) with 0.1 % formic acid. The flow rate was set to 300 µL/min. Samples were injected using a LC TriPlus RSH autosampler (CTC Analytics, Switzerland) and an injection volume of 10 µL in triplicate. The mass spectrometer was equipped with an Ion Max heated electrospray ionization source (HESI-II) operated in negative ionization mode during the analysis of the leaf litter and in positive ionization mode during the suspect screening of face mask leachate. The tune settings for negative ionization mode used -2.7 kV for spray voltage, 250 °C capillary temperature, 45 a.u. for sheath gas, 10 a.u. for auxiliary gas, 0 a.u. for sweep gas, probe heater temperature at 400 °C, and S-lens at 25 a.u. The tune settings for positive ionization mode used 2.5 kV for spray voltage, 350 °C capillary temperature, 35 a.u. for sheath gas, 10 a.u. for auxiliary gas, 0 a.u. for sweep gas, probe heater temperature at 250 °C, and the S-lens at 50 a.u.

Data analysis

Suspect Screening

The data was processed using Compound Discoverer (ver. 3.3) using a tailored workflow for analysis of leachables. The suspect list “Extractables and Leachables HRAM Compound Database”, which comes pre-installed in the software, was used for the analysis. The suspect list was matched against the high-resolution accurate masses obtained from the raw data. The procedural blanks were used blank subtraction, where for a feature to be considered “real”, it needed to have a three times higher signal in the sample than in the procedural blank. For quality control, only compounds which gave a peak rating of 6 or higher in all replicates were considered “real” peaks. Online publicly available spectral databases mzCloud and Massbank were used for prioritization of features. After processing, all compounds with a match against the spectral databases were manually investigated to remove false positives. Compounds which gave a match were given a confidence level following the Schymanski levels (Schymanski *et al.*, 2014).

Leaf litter dissolved organic matter analysis

The data was extracted using Qual Browser where all mass spectrometer signals were summed up from the chromatogram (retention time 1 – 14 minutes). This was exported to a csv file which was then imported into MATLAB (ver. R2017b). For the accurate mass analysis, a matrix containing a large number of molecular formulas were created. The formulas contained the following ranges: Carbon 1 – 40; Hydrogen 4 – 90; Oxygen 0 – 35; Nitrogen 0 – 1; Sulfur 0 – 1; carbon 13 isotope 0 – 1. To restrict the number of molecular formulas to more realistic candidates the following restrictions were used: m/z is between 100 and 700; hydrogen to carbon ratio (H/C) was greater or equal to 0.3 and less than or equal to 2.4; oxygen to carbon ratio (O/C) was greater than or equal to 0 and less than or equal to 1; adding up nitrogen, sulfur, and carbon 13 isotopes must be less than or equal to 1; double bond equivalence minus oxygen must be less than or equal to 10.

The recorded m/z from the sample were matched against the computer generated m/z with a mass accuracy tolerance of 2 ppm. The code would find the lowest difference in mass between the observed mass and the theoretical masses and if it was lower than 2 ppm it was accepted as a real formula. The molecular formulas were divided into categories, based on the thresholds used in the work of Brock *et al.* (2020).

Results

*Plastics*

The suspect screening data yielded one hit of particular interest in mzCloud and MassBankEU spectral databases. The compound gave a high match factor of 92.8, 1,3-di-o-Tolylguanidine (DTG). The compound is used in plastic and rubber production and is under regulation from the European Chemicals Agency due to it being “toxic if swallowed and is harmful to aquatic life with long lasting effects” (*ECHA*, 2024). Studies performed on rats have shown that exposure to DTG is detrimental to health and may have teratogenic effects, causing malformed offspring (Ema, Fujii, *et al.*, 2006b; Ema, Kimura, *et al.*, 2006b). Additionally, the United States Environmental Protection Agency (EPA) has predicted using their toxicity prediction pipeline, ToxCast, that the compound may interact with and disrupt, the sonic hedgehog signaling molecule (SHH)(Williams *et al.*, 2017; U.S. Environmental Protection Agency). This gene has been shown to be “instrumental in patterning the early embryo” in the human genome (NCBI, 2024). It is however important to note that nitrile gloves that have been linked to exposure of DTG were used during sample preparation. The inclusion of a procedural blank, which was treated the same as the leaching samples, did not show any DTG peaks. This excludes contamination from sample preparation as the cause for the presence of DTG in the samples.

*Wood*

The resulting feature list was used to make a van krevelen diagram to visualize the spread of compounds in the chemical space of the leaf litter leachate (Fig S2). The van krevelen diagram is used to assess the relative oxygen to carbon ratio and hydrogen to carbon ratio of each annotated feature. This can further be used to divide the compounds into chemical classes, which can give insight into what type of compounds can be expected to leach out of leaf litter into water.

The annotated features of each category were counted and plotted into a bar plot. The chemical classes were based on the parameters indicated in the work by Brock *et al.* (2020). Of the annotated features, 47.7% were CRAM (carboxyl-rich alicyclic molecules), also defined as lignin, 23.3 % of the features were identified as carbohydrates, 14.1 % were identified as tannins, 9.5 % were identified as lipids, 5.0 % were identified as unsaturated hydrocarbons, and finally 0.4 % were identified as condensed aromatics (Fig S2).

Figure S10. (a) Van krevelen diagram and bar plot of the feature annotation of the leaf litter. (b) Bar plot containing the chemical classifications as determined from the molecular formulas.

(b)

(a)

**References**

Brock, O. *et al.* (2020) ‘Non-target screening of leaf litter-derived dissolved organic matter using liquid chromatography coupled to high-resolution mass spectrometry (LC-QTOF-MS)’, *European Journal of Soil Science*, 71(3), pp. 420–432.

*ECHA* (2024) *1,3-di-o-tolylguanidine*. Available at: https://echa.europa.eu/substance-information/-/substanceinfo/100.002.344 (Accessed: 24 July 2024).

Ema, M., Fujii, S., *et al.* (2006a) ‘Prenatal developmental toxicity study of the basic rubber accelerator, 1,3-di-*o*-tolylguanidine, in rats’, *Reproductive Toxicology*, 22(4), pp. 672–678. Available at: https://doi.org/10.1016/j.reprotox.2006.05.003.

Ema, M., Fujii, S., *et al.* (2006b) ‘Prenatal developmental toxicity study of the basic rubber accelerator, 1,3-di-o-tolylguanidine, in rats’, *Reproductive Toxicology*, 22(4), pp. 672–678. Available at: https://doi.org/10.1016/j.reprotox.2006.05.003.

Ema, M., Kimura, E., *et al.* (2006a) ‘Reproductive and developmental toxicity screening test of basic rubber accelerator, 1,3-di-*o*-tolylguanidine, in rats’, *Reproductive Toxicology*, 22(1), pp. 30–36. Available at: https://doi.org/10.1016/j.reprotox.2005.11.002.

Ema, M., Kimura, E., *et al.* (2006b) ‘Reproductive and developmental toxicity screening test of basic rubber accelerator, 1,3-di-o-tolylguanidine, in rats’, *Reproductive Toxicology*, 22(1), pp. 30–36. Available at: https://doi.org/10.1016/j.reprotox.2005.11.002.

NCBI (2024) *SHH - sonic hedgehog signaling molecule (human)*. Available at: https://pubchem.ncbi.nlm.nih.gov/gene/SHH/human (Accessed: 24 July 2024).

Schymanski, E.L. *et al.* (2014) ‘Identifying small molecules via high resolution mass spectrometry: communicating confidence’. ACS Publications.

U.S. Environmental Protection Agency (no date) *1,3-Di-o-tolylguanidine*, *Comptox Chemicals Dashboard.* Available at: https://comptox.epa.gov/dashboard/chemical/details/DTXSID2026606 (Accessed: 24 July 2024).

Williams, A.J. *et al.* (2017) ‘The CompTox Chemistry Dashboard: a community data resource for environmental chemistry’, *Journal of Cheminformatics*, 9(1), p. 61. Available at: https://doi.org/10.1186/s13321-017-0247-6.
